# Role of glutamate transporter 1 in the attenuation of alcohol intake

**DOI:** 10.3389/fnins.2014.00200

**Published:** 2014-07-17

**Authors:** Youssef Sari

**Affiliations:** Department of Pharmacology, College of Pharmacy and Pharmaceutical Sciences, University of ToledoToledo, OH, USA

**Keywords:** EAAT2, GLT1, glutamate, alcohol dependence, P rats

Evidence demonstrated that many aspects of drug abuse and dependence involve changes in glutamate neurotransmission. Neuroadaptations of the glutamatergic system are critical in alcohol dependence, tolerance and withdrawal (Krystal et al., 2003; Backstrom and Hyytia, 2005; Cowen et al., 2005; Olive et al., 2005; Hodge et al., 2006; Bird et al., 2008; Kapasova and Szumlinski, 2008; Besheer et al., 2010). One of the selective effects of alcohol has been determined to be the inhibition of glutamatergic neurotransmission by antagonizing N-methyl-D-aspartate (NMDA) receptors (Grant et al., 1990; Chen et al., 1997). Furthermore, one of the effects of chronic alcohol exposure is the upregulation of NMDA receptors that results from chronic inhibition of glutamate transmission as a compensatory mechanism (Grant et al., 1990; Sanna et al., 1993; Snell et al., 1996; Chen et al., 1997). In addition, the effects of alcohol withdrawal have been found to be associated with increased extracellular glutamate levels in the striatum (Rossetti and Carboni, 1995), and enhanced NMDA sensitivity in the nucleus accumbens (NAc) of alcohol dependent rats (Siggins et al., 2003). Importantly, studies have reported that alcohol exposure affects glutamate transport and glutamate transmission (Smith, 1997; Smith and Weiss, 1999; Othman et al., 2002).

Although the neurocircuitry of the glutamatergic system is not fully defined, it has been suggested that the prefrontal cortex (PFC) (Goldstein and Volkow, 2002) and the NAc (Childress et al., 1999) play a critical role in drug reinforcement. These brain regions receive input from midbrain dopaminergic neurons, and all major drugs of abuse, including alcohol, increase forebrain dopamine transmission (Berridge and Robinson, 1998; Kalivas, 2004). The important roles of these glutamatergic projections from the PFC to the NAc and the ventral tegmental area (VTA) have been observed in neuroimaging studies performed during craving periods in several paradigms for commonly abused drugs such as alcohol, cocaine, methamphetamine, heroin and nicotine (Childress et al., 1999; Goldstein and Volkow, 2002). Moreover, glutamatergic projections from the PFC to the NAc are also important in the expression of addictive behaviors, and are the primary driver of drug abuse, including alcohol (for review see Kalivas, 2004; Rao and Sari, 2012).

Glutamate neurotransmission is regulated by several glutamate transporters. Among them, glutamate transporter 1 (GLT1, its human homolog is excitatory amino acid transporter 2, EAAT2) regulates the majority of extracellular glutamate (Robinson, 1998; Danbolt, 2001). GLT1 is present in the brain in two splice variant isoforms such as GLT1a and GLT1b (Chen et al., 2002, 2004; Berger et al., 2005). It has been reported that GLT1a is predominantly localized in neurons and astrocytes, and GLT1b is localized in astrocytes (Berger et al., 2005; Holmseth et al., 2009). Both isoforms regulate extracellular glutamate at the synaptic clefts. Our central question in our laboratory was whether we could increase the expression of GLT1 level in rat brains exposed to alcohol, and further determine the effects of this increase in alcohol intake. The increase in the expression of GLT1 can lead to the reduction of the amount of glutamate available to activate neurons in central reward brain regions, and thus decrease the craving initiated by it.

Studies have tested more than 1040 FDA-approved drugs to determine target compounds that may have effects in upregulating the expression of GLT1 (Rothstein et al., 2005). Rothstein et al. (2005) have found that among several β-lactam antibiotics, ceftriaxone was the potent drug that has an upregulatory effect in the expression of GLT1. This drug has been used to treat meningitis and is in phase III clinical trials for the treatment of Amyotrophic Lateral Sclerosis. We further examined the ability of this drug to increase the level of GLT1 and thereby decrease the amount of extracellular glutamate available to activate addictive behaviors. Thus, elevation of the expression of GLT1 might be associated with reduction in alcohol consumption as well as attenuation of relapse to alcohol intake.

We have used the animal model of alcohol-preferring (P) rats to measure the effectiveness of ceftriaxone in reducing alcohol consumption. These rats naturally prefer drink alcohol to plain water. After 5 weeks of a constant free choice of alcohol, the rats develop alcohol dependence. We administered ceftriaxone to the rats each day for 5 days and measured their alcohol consumption. P rats treated with ceftriaxone reduced their alcohol intake as compared to rats treated with physiological saline solution (Sari et al., 2011). This reduction in alcohol drinking was associated with increased GLT1 level in central reward brain regions, including the PFC and NAc. As shown in Figure 1 (Upper panel) that prior ceftriaxone treatment the level of GLT1 is low and the extracellular glutamate is higher in the NAc. Importantly, after ceftriaxone treatment the level of GLT1 is higher and in turn extracellular glutamate was lower. This suggests the beneficial effect of ceftriaxone in regulating glutamate homeostasis.

**Figure 1:**
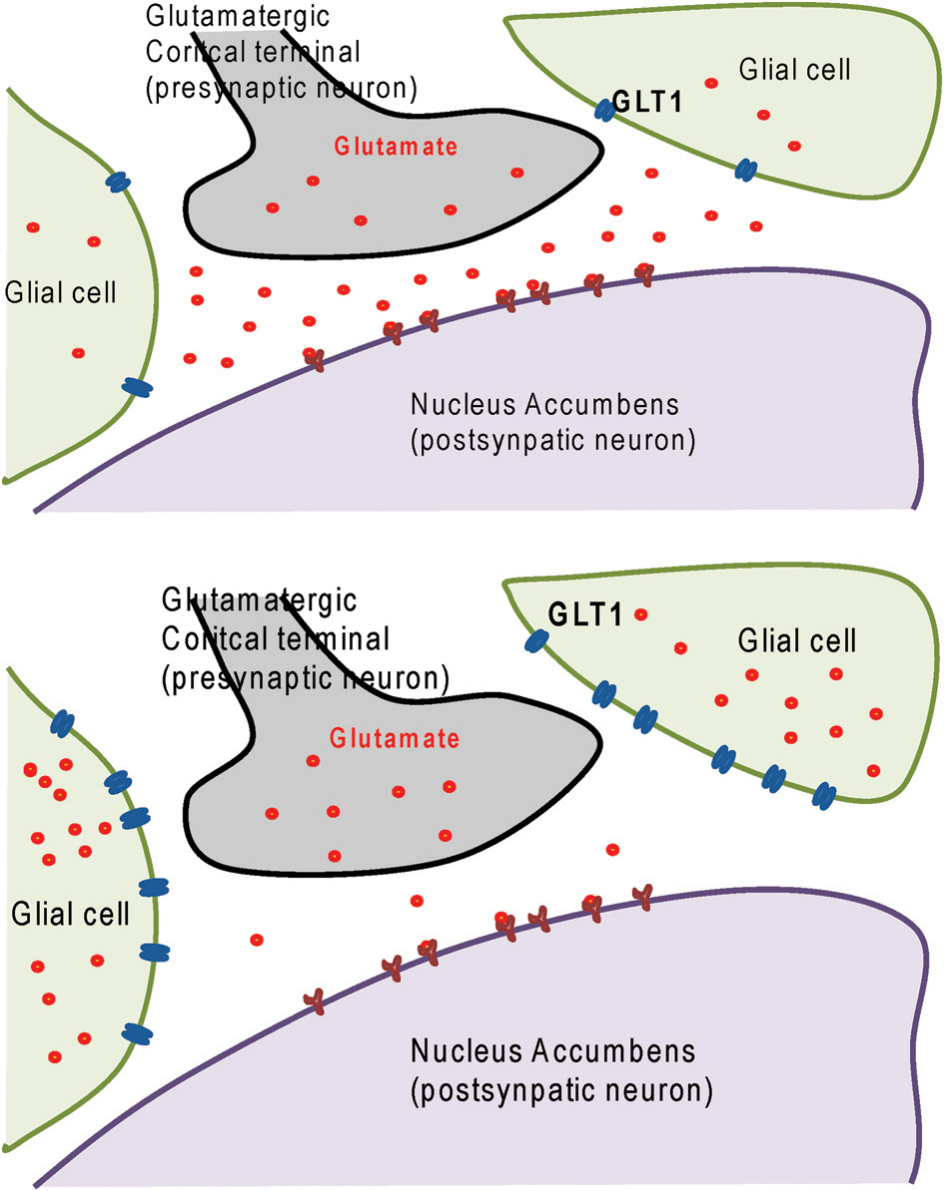
**Upper panel:** diagram shows glutamatergic cortical terminal in contact with postsynaptic neuron in nucleus accumbens surrounded by glial cells expressing glutamate transporter 1 (GLT1). **Lower panel:** diagram shows increased of GLT1 level in glial cells and decreased synaptic glutamate concentration as a consequence of treatment with GLT1 upregulator such as ceftriaxone and GPI-106.

We have also tested another drug, namely GPI-1046, which is a Neuroimmunophilin known to upregulate GLT1 level (Ganel et al., 2006). This compound has been shown to be effective in reducing alcohol intake (Sari and Sreemantula, 2012). This study from our laboratory demonstrated that this reduction was associated in part with elevation of GLT1 level in PFC and NAc.

We recently reported that ceftriaxone administration in male P rats is effective after 14 weeks of ethanol drinking paradigm and has a long-lasting effect after 10 days post-treatment (Rao and Sari, 2014). Furthermore, ceftriaxone treatment during deprivation period after 5 weeks of ethanol consumption attenuated relapse to alcohol drinking in male P rats (Qrunfleh et al., 2013). We also have shown that ceftriaxone attenuated the maintenance of ethanol rather than the acquisition of alcohol in female P rats (Sari et al., 2013). Importantly, our study with ceftriaxone demonstrated that elevation of GLT1 level in PFC and NAc can lead to the attenuation of relapse to cocaine-seeking behavior (Sari et al., 2009). Cocaine is an addictive substance that shares about the same neurocircuitry as alcohol. The neurochemistry is different in alcohol and cocaine addiction, but glutamate plays a similar role in both cases. These findings provide a solid foundation for targeting GLT1 for the treatment of drugs abuse, including alcohol.

It is noteworthy that studies from our laboratory identified another glial protein involved in glutamate homeostasis. This protein termed cysteine/glutamate exchanger transporter (xCT) was found downregulated in animal consumed alcohol for 5 weeks (Alhaddad et al., 2014). Importantly, this later study demonstrated that ceftriaxone reversed this downregulation of GLT1 level in NAc and PFC. Furthermore, we have demonstrated that ceftriaxone upregulated GLT1 in amygdala, PFC and NAc even when the rats consumed alcohol for 14 days (Rao and Sari, 2014). Studies also have shown that ceftriaxone upregulated xCT level in relapse to cocaine seeking (Knackstedt et al., 2010). These findings suggest xCT as another target protein for the treatment of drug abuse, including alcohol.

Furthermore, studies have shown that adenosine plays an important role in regulating the activity of neurons and controlling neurotransmitters, including GABA, glutamate and dopamine (for review see Nam et al., 2012). Alcohol has been shown to increase extracellular adenosine levels, which in turn regulate the ataxic and hypnotic/sedative (somnogenic) effects of alcohol. Adenosine signaling is also involved in the homeostasis of major inhibitory (GABA) and excitatory (Glutamate) neurotransmission through neuron-glial interactions. These interactive mechanisms regulate the effects of alcohol and sleep (for review see Nam et al., 2012). Furthermore, adenosine exerts its function through several adenosine receptors and regulates glutamate levels in the brain, which modulate alcohol dependence and sleep patterns.

Alcohol abuse and dependence continue to be significant public health concerns. Thus, a better understanding of their neurobiology would facilitate the development of interventions targeting prevention and/or treatment of these major health issues. Here, we focused on the glutamatergic system as therapeutic target for the treatment of alcohol dependence. We have identified potential therapeutic compounds that may have beneficial effects for treating alcohol addiction. We believe that a focus on the glutamatergic system as a prime candidate for mediating drug and alcohol dependence.

## Conflict of interest statement

The author declares that the research was conducted in the absence of any commercial or financial relationships that could be construed as a potential conflict of interest.
